# Preventing ovarian aging: from redox-targeted strategies to extracellular vesicle-based therapies

**DOI:** 10.3389/fragi.2026.1707614

**Published:** 2026-02-24

**Authors:** Chiara Camerano Spelta Rapini, Camila Cecilia Rojo-Fleming, Chiara Di Berardino, Alessia Peserico, Giulia Capacchietti, Umberto Tosi, Nicola Bernabò, Mauro Mattioli, Barbara Barboni

**Affiliations:** 1 Department of Bioscience and Technology for Food, Agriculture and Environment, University of Teramo, Teramo, Italy; 2 Institute of Biochemistry and Cell Biology (CNR-IBBC/EMMA/Infrafrontier/IMPC), National Research Council, Monterotondo Scalo, Rome, Italy

**Keywords:** extracellular vesicles (EVs), mitochondrial dysfunction, ovarian aging, ovarian dysfunction, oxidative stress

## Abstract

Ovarian aging is increasingly recognized as a dynamic and modifiable process influenced by oxidative stress, mitochondrial dysfunction, and chronic inflammation. This review outlines the mechanisms by which environmental and lifestyle factors, such as smoking, high-fat diets, endocrine-disrupting chemicals, and micro- and nanoplastics (MNPs), contribute to accelerated ovarian decline and premature reproductive senescence. The distinction between physiological aging and pathological processes such as “inflamm-aging” is discussed, with particular attention to redox imbalance and mitochondrial impairment as key drivers of follicular depletion and endocrine dysfunction. Insights from experimental models of premature ovarian insufficiency and polycystic ovary syndrome are summarized to illustrate the role of reactive oxygen species and oxidative damage. Current antioxidant-based strategies aimed at delaying ovarian aging are reviewed, including melatonin, N-acetylcysteine, coenzyme Q10, polyphenols, and vitamins C and E. Particular emphasis is placed on the emerging potential of stem cell-derived extracellular vesicles (EVs) as a novel, cell-free therapeutic approach. Preclinical evidence suggests that EVs can reduce oxidative stress, support mitochondrial function, and restore ovarian physiology. Overall, the review highlights how redox-targeted and EV-based interventions may offer promising avenues to preserve ovarian function and extend reproductive healthspan.

## 1 Introduction

Ovarian function plays a crucial role not only in reproductive competence but also in maintaining systemic homeostasis, impacting cardiovascular (Roeters van Lennep et al., 2016), skeletal (Gallagher, 2007), and cognitive health (Sochocka et al., 2023; Hamoda and Sharma, 2024; Cavalcante et al., 2023). While physiological ovarian aging represents a gradual and predictable process, its pathological acceleration, often mediated by oxidative stress and chronic inflammation, poses a disproportionate threat to women’s health, particularly when it occurs prematurely (Kobayashi et al., 2025; Zhang et al., 2025; Salminen et al., 2012; Mu et al., 2025).

What differentiates physiological ovarian aging from its pathological counterpart is not solely the rate of follicular depletion, but rather the specific nature and origin of the underlying molecular insults. Oxidative stress, often triggered by environmental (Camerano Spelta Rapini et al., 2024), dietary (Di Berardino et al., 2024; Di Berardino et al., 2022; Gonnella et al., 2022), or metabolic factors (Camerano Spelta Rapini et al., 2024; Xue et al., 2024), plays a central role in undermining ovarian resilience. This has led to the conceptual emergence of “inflamm-aging,” a model that reframes ovarian aging as a systemic, modifiable process rather than an inevitable outcome of chronology (Yan et al., 2022; Li X. et al., 2023; Isola et al., 2024a).

Premature ovarian insufficiency (POI), previously referred to as premature ovarian failure or premature menopause, affecting approximately 1% of women under 40, is not only a reproductive crisis but a sentinel event with profound long-term health consequences, ranging from cardiovascular and skeletal complications to cognitive decline (Roeters van Lennep et al., 2016; Gallagher, 2007; Sochocka et al., 2023; Hamoda and Sharma, 2024). Yet, despite the growing recognition of oxidative stress as a mechanistic driver, current therapies fail to reverse or significantly delay this trajectory (Yan et al., 2022).

In this context, the therapeutic potential of stem cell-derived EVs is particularly compelling. As a regenerative approach that does not require transplanting live cells, EVs therapy may directly target the root mechanisms of ovarian dysfunction, mitochondrial failure, reactive oxygen species (ROS) accumulation, and inflammation, rather than merely alleviating downstream symptoms (Luo et al., 2024; Feng et al., 2020; Ling et al., 2017; Ling et al., 2019; Keshtkar et al., 2018; Wang et al., 2017).

The review examines ovarian aging in relation to oxidative stress and redox imbalance, highlighting how environmental, metabolic, and dietary factors accelerate follicular loss and endocrine dysfunction. Finally, it examines redox-targeted interventions and emerging EVs-based strategies for preserving ovarian function. Accordingly, the review follows a progression from mechanisms to modifiers, disease models, and therapeutic strategies.

## 2 Physiological ovarian aging vs. inflamm-aging

Ovarian aging is often viewed as an unavoidable, time-bound phenomenon characterized by the depletion of primordial follicles and a steady decline in oocyte quality (Yan et al., 2022; Yang L. et al., 2020). This process, while biologically programmed, is shaped by complex interactions among hormonal fluctuations, metabolic shifts, and cellular senescence. However, emerging data challenge the idea that aging alone dictates ovarian decline, suggesting that inflammatory and oxidative processes act as accelerants, disrupting the finely tuned endocrine and cellular architecture of the ovary (Briley et al., 2016; Isola et al., 2024b; Orisaka et al., 2023).

The term inflamm-aging refers to the chronic activation of inflammatory pathways that accompanies aging and contributes to tissue dysfunction (Huang et al., 2019). Unlike physiological aging, which unfolds over decades, inflamm-aging is characterized by the activation of pro-inflammatory mediators, such as IL-6, TNF-α, and chemokines, which can induce follicular atresia, compromise steroidogenesis, and hasten menopause (Yan et al., 2022). This distinction is not merely semantic: it points to an opportunity for intervention through modulation of oxidative and immune signaling pathways (Zhang et al., 2025; Isola et al., 2024a; Smits et al., 2023; Ju et al., 2024).

By contrasting these trajectories, we can identify potential therapeutic leverage points, shifting focus from symptomatic management to upstream prevention and biological restoration.

### 2.1 From reproductive cycle to menopause

The female reproductive lifespan is governed by the hypothalamic-pituitary-ovarian (HPO) axis, which orchestrates cyclical hormonal dynamics essential for ovulation, endometrial remodeling, and fertility (Spaziani et al., 2021). With age, the progressive depletion of the ovarian follicular pool leads to declining levels of estradiol and inhibins, resulting in a compensatory rise in gonadotropins such as FSH (Spaziani et al., 2021; Park et al., 2021; Allshouse et al., 2018). This process ultimately culminates in menopause, the permanent cessation of ovarian endocrine and reproductive function, typically around the age of 51 (Peacock et al., 2025).

Perimenopause precedes this transition and is characterized by fluctuating hormone levels and irregular menstrual cycles, often accompanied by vasomotor symptoms, mood disturbances, and sleep disruption (Coborn et al., 2022; Duralde et al., 2023). While this physiological progression is well-documented, deviations from the expected timeline, particularly in premature presentations, warrant deeper investigation into underlying molecular mechanisms. In such cases, factors like oxidative stress, chronic inflammation, and immune dysregulation may contribute to accelerated follicular depletion and endocrine failure (Hamoda and Sharma, 2024), forming the basis for the mechanisms explored in the following sections.

### 2.2 Premature ovarian insufficiency (POI)

POI is defined as the loss of ovarian function before age 40, leading to hypoestrogenism, elevated FSH levels, and infertility (Hamoda and Sharma, 2024). Affecting ∼1% of women, its implications extend beyond reproductive concerns, encompassing cardiovascular morbidity, skeletal fragility, and neurocognitive risk (Roeters van Lennep et al., 2016; Gallagher, 2007; Sochocka et al., 2023; Liang C. et al., 2023).

Although hormone replacement therapy (HRT) remains the standard of care, it neither restores ovarian reserve nor addresses the underlying molecular damage. A more comprehensive understanding of POI pathogenesis, particularly the contribution of oxidative stress, could support the development of disease-modifying therapies, moving beyond symptomatic management.

### 2.3 Mechanisms underlying POI

POI can arise from a variety of mechanisms, although idiopathic cases, those with no clearly identifiable cause, represent the vast majority, accounting for 85%–90% of instances (Kapoor, 2023). Among the known causes are genetic abnormalities, such as Turner syndrome (Bollig et al., 2023) and Fragile X (Man et al., 2017) premutation carriers, which can compromise ovarian development and function (Ebrahimi and Akbari Asbagh, 2011). Autoimmune disorders, including autoimmune thyroiditis (Meling Stokland et al., 2022), Addison’s disease, and systemic lupus erythematosus, may also disrupt ovarian activity through immune-mediated damage (Szeliga et al., 2021). Certain metabolic conditions like galactosemia (Sozen et al., 2019; Thakur et al., 2018), which involve enzyme deficiencies, have similarly been implicated. Infectious diseases, particularly viral infections such as mumps oophoritis or chronic infections like tuberculosis, can directly impair ovarian tissue (Panay and Kalu, 2009; Goswami and Conway, 2005). Additionally, iatrogenic factors, most commonly chemotherapy or radiotherapy, frequently result in POI (Sklar, 2005; Tham et al., 2007; Oktem and Oktay, 2007). In many cases, the onset of POI appears to reflect a complex interplay between genetic predisposition and environmental exposures (Sapre and Thakur, 2014; Gold et al., 2013), often culminating in oxidative stress-mediated follicular damage. Elevated ROS can injure oocytes and granulosa cells, accelerating atresia and depleting the ovarian reserve. This redox imbalance, whether triggered by toxins, inflammation, or mitochondrial dysfunction, is increasingly recognized as a prime target for interventions aimed at preserving ovarian function (Yan et al., 2022; Jadeja et al., 2021; Agarwal et al., 2008; Behrman et al., 2001). Recognizing these underlying mechanisms is crucial for effective clinical intervention and management, aiming to preserve fertility and mitigate health risks related to early estrogen depletion.

#### 2.3.1 Cellular senescence and release of SASP factors

Senescence in granulosa and stromal cells leads to the release of pro-inflammatory and matrix-degrading molecules collectively termed the senescence-associated secretory phenotype (SASP) (Hense et al., 2024; Harada, 2024). This secretome, rich in cytokines (IL-6, TNF-α), chemokines (CXCL1, CCL2), and MMPs, acts in a paracrine fashion to perpetuate tissue damage, inflammation, and further oxidative stress (Li X. et al., 2023; Hense et al., 2024; Babayev and Duncan, 2022).

Importantly, SASP may not merely be a byproduct of aging, but a driver of it, contributing to a self-reinforcing loop of tissue deterioration, follicular dysfunction, and reproductive failure.

#### 2.3.2 Impaired mitochondrial bioenergetics and reduced antioxidant defenses

Growing and maturing oocytes, as well as primordial oocytes are energetically demanding cells reliant on efficient mitochondrial oxidative phosphorylation (OXPHOS) (Smits et al., 2023; Bao et al., 2024; Rodríguez-Nuevo et al., 2022; Zhang et al., 2022). Aging impairs mitochondrial biogenesis, disrupts the electron transport chain, and reduces ATP output, changes that correlate with reduced developmental competence and increased follicular atresia (Bao et al., 2024; Iwata et al., 2011; Liang et al., 2020).

Simultaneously, the aging ovary experiences a decline in key antioxidant systems, SOD, catalase and GSH, rendering it more vulnerable to ROS-induced damage (Bao et al., 2024; Liang et al., 2020). Preclinical studies suggest that mitochondrial-targeted antioxidants (e.g., CoQ10, NR) can partially restore bioenergetics and follicular integrity (Bao et al., 2024).

#### 2.3.3 Chronic activation of NF-κB and suppression of Nrf2 pathways

The NF-κB/Nrf2 axis represents a molecular fulcrum between inflammation and oxidative defense. In aging ovaries, chronic NF-κB activation sustains a pro-inflammatory state, while suppressed Nrf2 signaling diminishes the expression of detoxifying and antioxidant genes (Li X. et al., 2023; Hense et al., 2024). This imbalance fosters an environment of unmitigated oxidative injury, accelerating follicular decline. In contrast, the Nrf2 pathway serves a protective role by activating antioxidant response elements (ARE) and upregulating genes encoding antioxidant enzymes (Li X. et al., 2023).

Experimental inhibition of NF-κB or activation of Nrf2 has been shown to reduce ovarian ROS levels, preserve follicular structure, and improve functional outcomes suggesting this pathway as a promising therapeutic target.

**FIGURE 1:**
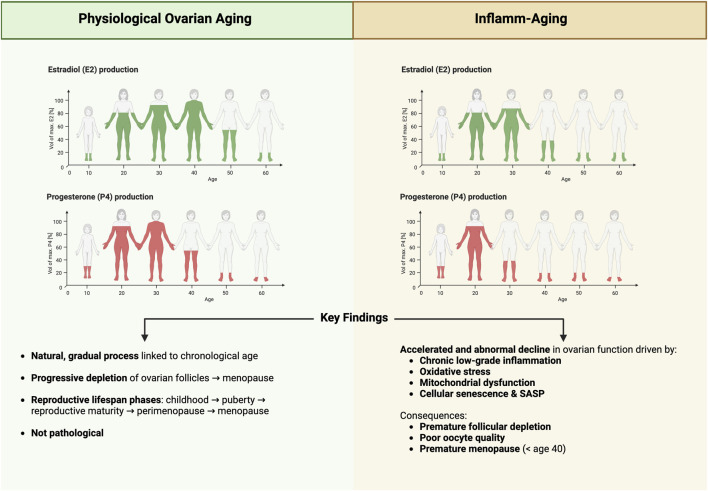
Comparison between Physiological Ovarian Aging and Inflamm-Aging. Schematic representation of estradiol (E2) and progesterone (P4) production across the female lifespan under physiological conditions (left) and in the context of inflamm-aging and POI (right). Physiological ovarian aging follows a gradual, time-dependent decline in hormonal output and follicular reserve, leading to natural menopause around age 50. In contrast, inflamm-aging is characterized by chronic low-grade inflammation, oxidative stress, mitochondrial dysfunction, and cellular senescence, which accelerate ovarian decline. This pathological trajectory results in reduced hormone production and may lead to POI before the age of 40. Created in https://BioRender.com.

A schematic summary of the concepts discussed in this section is illustrated in Figure 1, highlighting the differences between physiological ovarian aging and the pathological trajectory driven by inflamm-aging.

## 3 Environmental and lifestyle drivers of accelerated ovarian aging

A growing body of evidence supports the notion that menopause timing is not solely genetically determined but is significantly influenced by lifestyle and environmental exposures. Behaviors such as cigarette smoking (Konstantinidou et al., 2021; Ruder et al., 2009), consumption of high-fat diets (Di Berardino et al., 2024; Di Berardino et al., 2022; Yan et al., 2020), and contact with endocrine disruptors (e.g., MNPs, (Camerano Spelta Rapini et al., 2024), BPA (Wang et al., 2014; Vandenberg et al., 2007) can modulate oxidative stress, mitochondrial integrity, and inflammatory responses, ultimately shaping ovarian lifespan.

The next section explores these modifiable factors in detail, evaluating both epidemiological trends and mechanistic evidence linking them to accelerated reproductive aging.

### 3.1 Tobacco smoke: a potent gonadotoxin

Among lifestyle factors, cigarette smoking stands out as one of the most consistent accelerators of ovarian aging. Epidemiological studies show that smokers, both current and former, experience menopause up to 4 years earlier than non-smokers, with a clear dose-response pattern in heavy smokers (Yan et al., 2022; Pappas, 2011). But beyond correlations, the mechanistic impact of tobacco smoke is particularly striking: it delivers a toxic cocktail of over 7,000 chemicals, many with gonadotoxic, mutagenic, and endocrine-disrupting effects (Pappas, 2011; Zeng et al., 2023).

Key agents like polycyclic aromatic hydrocarbons (PAHs) bind to aryl hydrocarbon receptors (AhR) in ovarian cells, activating apoptotic cascades and promoting follicular depletion. At the same time, smoking induces systemic and local oxidative stress, impairing mitochondrial integrity and disrupting estrogen synthesis by downregulating aromatase and steroidogenic transport proteins. These mechanisms collectively reduce estradiol levels and destabilize the hypothalamic-pituitary-ovarian axis, resulting in irregular cycles and a more symptomatic perimenopause (Matikainen et al., 2001).

Lower anti-Müllerian hormone (AMH) levels in smokers further support the notion that tobacco use accelerates the decline in ovarian reserve (Plante et al., 2010; Dólleman et al., 2013). In short, cigarette smoking acts as a multifactorial disruptor, combining cytotoxicity, oxidative damage, hormonal imbalance, and apoptotic acceleration, to shorten reproductive lifespan and hasten the transition to menopause.

### 3.2 Endocrine-disrupting chemicals and MNPs: silent intruders

Exposure to endocrine-disrupting chemicals, such as phthalates, bisphenol A (BPA), polybrominated diphenyl ethers (PBDEs), and perfluoroalkyl substances (PFAS), has been associated with accelerated ovarian aging and POI. Several cross-sectional and longitudinal studies report that higher systemic levels of endocrine-disrupting chemicals correlate with reduced antral follicle count, earlier loss of ovarian function, and altered hormone levels (Grindler et al., 2015). Mechanistically, these compounds induce oxidative stress, inflammation, and apoptosis in granulosa cells, while interfering with steroidogenesis and folliculogenesis.

More recently, MNPs, especially polystyrene-derived particles, have emerged as an additional environmental concern. These particles, found in food, water, and air, have been detected in human ovarian follicular fluid, raising alarms over their potential to impair follicle viability and hormone secretion. Experimental models demonstrate that MNPs exposure leads to increased apoptosis, granulosa cell dysfunction, disruption of estrous cyclicity, and impaired ovarian development (Camerano Spelta Rapini et al., 2024). Additionally, MNPs may act as “Trojan horses” by facilitating the delivery of toxic endocrine disruptors chemicals such as phthalates, PFAS, and BPA into sensitive ovarian tissues, further amplifying their detrimental effects.

Together, these findings underscore the critical role of modifiable environmental and behavioral exposures in shaping the trajectory of ovarian aging and reproductive lifespan.

#### 3.2.1 Mechanistic insights: endocrine disruptors and MNPs

At the mechanistic level, endocrine disruptors chemicals and MNPs converge on several key pathways that impair ovarian health. Rather than acting through distinct mechanisms, these exposures amplify oxidative stress-driven mitochondrial dysfunction, genomic instability, and inflammatory signaling within the ovarian microenvironment. Excessive ROS damage oocyte DNA, impair mitochondrial function, and drive granulosa cell apoptosis, hallmarks of accelerated ovarian aging (Camerano Spelta Rapini et al., 2024; Srnovršnik et al., 2023).

Endocrine disruptors chemicals such as phthalates and BPA bind to estrogen receptors (ERα/ERβ), disrupting hormone signaling and impairing the hypothalamic-pituitary-ovarian axis. They also downregulate steroidogenic enzymes including aromatase, StAR, and 3β-HSD, leading to reduced estradiol synthesis and compromised follicular development (Camerano Spelta Rapini et al., 2024; Srnovršnik et al., 2023).

MNPs exacerbate these effects by entering ovarian cells through endocytosis or paracellular transport. Once internalized, they activate inflammatory pathways (e.g., NF-κB) and promote the release of cytokines like IL-6 and TNF-α. Simultaneously, they upregulate apoptotic markers (e.g., Bax, caspase-3) and downregulate survival signals (e.g., Bcl-2), mimicking processes seen in senescent or damaged ovaries (Camerano Spelta Rapini et al., 2024; Srnovršnik et al., 2023).

Critically, MNPs enhance the delivery of endocrine disruptor chemicals and heavy metals to ovarian tissue, intensifying their harmful effects. This combined “Trojan horse” mechanism fosters a toxic intraovarian environment marked by inflammation, oxidative stress, and premature follicular depletion (Camerano Spelta Rapini et al., 2024; Srnovršnik et al., 2023).

**FIGURE 2:**
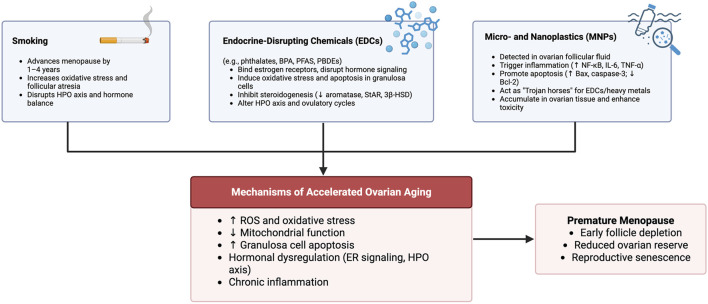
Environmental Risk Factors Contributing to Accelerated Ovarian Aging and POI. This figure illustrates the key environmental contributors to premature ovarian aging: smoking, endocrine-disrupting chemicals, and MNPs. These factors act through common mechanisms, including increased oxidative stress, mitochondrial dysfunction, granulosa cell apoptosis, hormonal disruption, and chronic inflammation. Together, these biological insults accelerate follicular depletion and lead to POI, characterized by early loss of ovarian reserve and reproductive senescence. Created in https://BioRender.com.

To visually summarize the shared mechanisms through which lifestyle and environmental exposures contribute to ovarian aging, the following Figure 2 highlights the key factors involved and their downstream effects leading to POI.

### 3.3 High-fat diet: metabolic assault on the ovary

The modern rise in high-fat diet (HFD) consumption has been strongly associated with declining female reproductive health, particularly through its detrimental effects on ovarian function and follicular dynamics. Emerging evidence indicates that excessive dietary lipid intake compromises ovarian resilience by triggering a cascade of metabolic disturbances that impair cellular homeostasis within the follicular environment. Specifically, HFDs contribute to lipid accumulation in non-adipose ovarian cells, promote lipotoxicity, induce chronic low-grade inflammation, and elevate ROS, ultimately disrupting oocyte development and fertility outcomes (Di Berardino et al., 2024; Di Berardino et al., 2022; Gonnella et al., 2022).

Understanding the mechanisms through which HFD undermines ovarian physiology is critical, as they extend beyond simple caloric excess to involve complex metabolic, oxidative, and inflammatory pathways. The following sections explore these mechanisms in detail, focusing on lipid-induced toxicity, inflammation-driven oxidative stress, and dysregulated fatty acid metabolism, and their collective impact on ovarian cell viability, follicular development, and reproductive potential.

#### 3.3.1 Lipid accumulation and lipotoxicity in ovarian cells

HFD, as well as stress or other conditions of negative energy imbalance, promotes lipid accumulation in ovarian non-adipose cells—including granulosa, cumulus, and theca cells, as well as oocytes—ultimately leading to lipotoxicity. This phenomenon is characterized by increased intracellular triglyceride content, endoplasmic reticulum (ER) stress, mitochondrial dysfunction, and apoptotic cell death (Di Berardino et al., 2022; Wu et al., 2010). *In vivo*, HFD represents the principal experimental model used to study ovarian lipotoxicity. Consistent with the reprotoxic effects of excessive lipolysis, long-term exposure to HFD in mice results in increased lipid droplet accumulation within oocytes and cumulus–oocyte complexes (COCs), reduced mitochondrial membrane potential, upregulated ER stress markers (ATF4, GRP78), and increased apoptosis in granulosa cells, culminating in decreased ovulation and fertilization rates (Wu et al., 2010).

Data from human follicular fluid confirm higher triglyceride levels in women with obesity, suggesting similar lipotoxic stress occurs clinically (Wu et al., 2010). Other studies have shown that HFD-driven lipid overload in follicular environments disrupts somatic-germ cell interactions, initiating mitochondrial and ER dysfunction within the follicle (Di Berardino et al., 2022; Gonnella et al., 2022). Overall, HFD-mediated lipid accumulation induces lipotoxic stress pathways, ER stress, mitochondrial injury, and apoptosis, impairing granulosa cell viability and oocyte competence.

#### 3.3.2 Metabolic inflammation and ROS production

Metabolic inflammation, characterized by adipokine dysregulation and systemic insulin resistance, is a hallmark of HFD and a key driver of ROS overproduction in ovarian tissue. HFD increases circulating levels of leptin and insulin, triggering local inflammatory responses, upregulation of pro-inflammatory cytokines such as TNF-α and IL-6, and activation of the NF-κB signaling pathway (Di Berardino et al., 2024; Di Berardino et al., 2022).

These inflammatory signals enhance ROS generation through both NADPH oxidases and mitochondrial electron transport chain dysfunction. The cumulative effect is elevated oxidative stress within ovarian follicles, as evidenced by increased malondialdehyde (MDA) levels in ovarian homogenates of HFD-fed murine models (Yan et al., 2020).

Different studies underscored the role of adipose-derived inflammatory mediators in driving oxidative stress under HFD conditions, linking them to follicular dysfunction and a reduced ovarian reserve (Di Berardino et al., 2024; Di Berardino et al., 2022; Gonnella et al., 2022).

Additional studies also report that chronic inflammation impairs key intracellular signaling cascades, such as PI3K/Akt and mTOR, in granulosa cells, thereby promoting ROS accumulation and impairing follicular maturation (Di Berardino et al., 2022; Gonnella et al., 2022).

Altogether, these findings indicate that HFD-induced metabolic inflammation is a central mechanism underlying ROS-mediated ovarian damage.

#### 3.3.3 Altered fatty acid metabolism affecting follicular growth

HFD disrupts ovarian fatty acid metabolism by upregulating fatty acid uptake and trafficking (increased CD36, FABP expression), impairing β-oxidation (reduced PPARα targets, CPT1, ACOX), and promoting lipid storage via PPARγ-mediated FSP27/CIDE-C upregulation, leading to ectopic lipid accumulation, lipotoxicity, and oxidative stress compromising folliculogenesis at multiple stages (Di Berardino et al., 2022) (Yan et al., 2020; Yang et al., 2016).

In HFD-fed rodents, increased expression of fatty acid transporters (e.g., CD36) and lipid droplet-associated proteins (e.g., FSP27) contributes to abnormal lipid accumulation and oxidative stress in ovarian cells (Ofosu-Boateng et al., 2024). Additionally, maternal and post-weaning HFD exposures affect mitochondrial dynamics (e.g., MFN2, MFF), lower antioxidant defenses (SOD, catalase), and elevate oxidative stress markers such as MDA, leading to reduced primordial follicle counts and impaired folliculogenesis (Yan et al., 2020).

Dysregulation of key nutrient-sensing and longevity-related regulators, such as SIRT1 and FOXO3, has also been observed under HFD conditions. These alterations impair the primordial-to-primary follicle transition and reduce antral follicle development, likely through disrupted lipid metabolism and ROS buildup (Gonnella et al., 2022).

In summary, altered fatty acid metabolism under HFD conditions disrupts follicular growth both directly-via lipid overload and mitochondrial impairment, and indirectly, through oxidative and inflammatory pathways.

**FIGURE 3:**
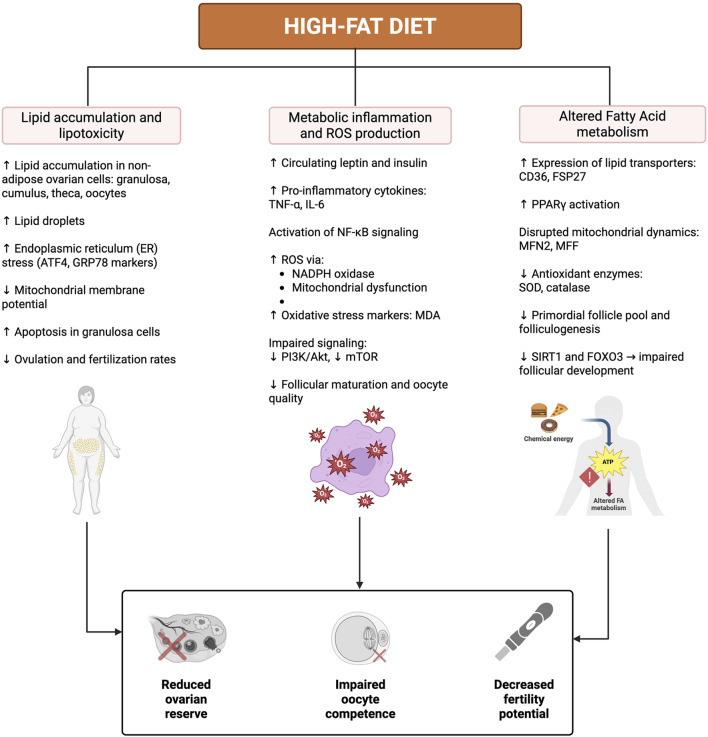
Mechanisms by Which High-Fat Diet Impairs Ovarian Resilience and Fertility. A high-fat diet (HFD) promotes lipid accumulation and lipotoxicity in ovarian cells, metabolic inflammation with increased ROS production, and disrupted fatty acid metabolism. These alterations lead to ER and mitochondrial stress, apoptosis, impaired intracellular signaling, and oxidative damage. Collectively, these mechanisms reduce ovarian reserve, compromise oocyte competence, and ultimately decrease fertility potential. Created in https://BioRender.com.

A visual summary of these pathways is illustrated in Figure 3.

### 3.4 Preliminary conclusion: converging path to reproductive senescence

**FIGURE 4:**
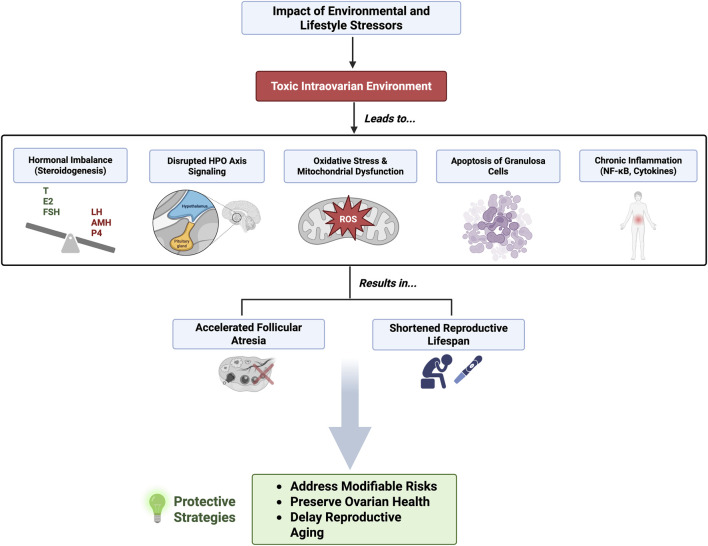
Impact of Environmental and Lifestyle Stressors on Ovarian Aging. Schematic representation of how environmental and lifestyle stressors contribute to a toxic intraovarian environment, leading to hormonal imbalance, mitochondrial dysfunction, inflammation, and ultimately accelerated follicular atresia and shortened reproductive lifespan. Protective strategies may help preserve ovarian health and delay reproductive aging. Created in https://BioRender.com.

Although environmental stressors may differ in origin, ranging from lifestyle factors to pollutants, they tend to converge on a limited number of shared pathophysiological mechanisms that drive reproductive aging. A central feature among these is oxidative stress, which impairs mitochondrial function and contributes to cellular damage within the ovary. In parallel, disruptions in hormonal homeostasis, particularly in steroidogenesis, further compromise follicular development and oocyte quality. These alterations are often accompanied by increased apoptosis of granulosa and other somatic ovarian cells, reducing the ovarian reserve. Additionally, persistent activation of inflammatory pathways, such as NF-κB signaling and the release of proinflammatory cytokines, sustains a chronic inflammatory state within the ovarian microenvironment. Compounding these effects is the disruption of neuroendocrine signaling along the hypothalamic-pituitary-ovarian (HPO) axis, which further destabilizes reproductive function. Collectively, these factors contribute to a hostile intraovarian environment that accelerates follicular atresia and leads to a premature decline in reproductive potential. Understanding these converging mechanisms highlights the importance of identifying and mitigating modifiable environmental risks in order to preserve ovarian function and delay reproductive senescence in modern populations. Figure 4 provides a schematic representation of the cascade of intraovarian events triggered by environmental and lifestyle stressors, ultimately contributing to accelerated ovarian aging.

## 4 From oxidative exposure to ovarian disease: mechanisms and clinical manifestations

Oxidative stress is increasingly recognized as a critical pathogenic link in several ovarian disorders, linking environmental insults, metabolic dysregulation, and aging to impaired reproductive outcomes. The imbalance between ROS generation and antioxidant defense disrupts mitochondrial function, promotes granulosa cell apoptosis, and accelerates follicular atresia, ultimately compromising ovarian reserve. Rather than acting as a uniform pathogenic signal, redox imbalance manifests with distinct temporal dynamics and biological consequences depending on disease context. This section builds on the environmental and lifestyle exposures discussed earlier to explore how context-dependent alterations in redox homeostasis underlie specific ovarian pathologies and their clinical manifestations (Yan et al., 2022; Deer et al., 2025).

### 4.1 Polycystic ovary syndrome (PCOS): insulin resistance, chronic inflammation, and ROS

PCOS is characterized by insulin resistance, hyperandrogenemia, chronic low-grade inflammation, and heightened oxidative stress. ROS production is elevated in granulosa cells and circulating leukocytes, and systemic markers such as malondialdehyde (MDA) and myeloperoxidase (MPO) are increased in PCOS patients, particularly those with insulin resistance (Victor et al., 2016). In this setting, oxidative stress acts primarily as a persistent, low-grade metabolic stressor, arising from mitochondrial dysfunction and NADPH oxidase activation, and amplified by inflammatory cytokines (TNFα, IL-6). This chronic redox imbalance interferes with mitochondrial biogenesis, ATP synthesis, and redox homeostasis, thereby impairing follicular development and oocyte quality (Yan et al., 2024). These pathogenic mechanisms have direct clinical consequences and inform targeted therapeutic strategies. As an example, interventions such as N-acetylcysteine (NAC), a precursor to glutathione, have been shown to partially rescue metabolic imbalance, improve insulin sensitivity, and reduce ovulatory dysfunction (Fang et al., 2024).

### 4.2 POI: mitochondrial failure and apoptotic follicular loss

In contrast to PCOS, POI is characterized by a more abrupt and severe disruption of redox homeostasis, leading to accelerated follicular depletion. Excessive ROS accumulation, mitochondrial DNA damage, and impaired mitophagy contribute to compromised oocyte and granulosa cell survival. Women with POI exhibit lower mtDNA copy numbers, frequent mitochondrial gene mutations, and defective oxidative phosphorylation (Kakinuma and Kakinuma, 2023). Systemic oxidative stress markers (d-ROMs, OSI), are markedly elevated reflecting a collapse of compensatory antioxidant defenses (Kakinuma and Kakinuma, 2023). Mitochondrial dysfunction, mediated by reduced SIRT1/SIRT3 signaling, leads to apoptotic follicular loss and diminished ovarian reserve (Guo et al., 2022). As treatments beyond conventional hormone replacement therapy, a new generation of experimental regenerative approaches for POI—including mitochondrial activation, *in vitro* follicle activation, stem cell and exosome therapies, biomaterial-based strategies, and intra-ovarian platelet-rich plasma infusion—holds promise for re-establishing oxidative balance and preserving ovarian function (Huang et al., 2022), although clinical translation remains limited.

### 4.3 Iatrogenic toxicity: ROS-induced granulosa cell death and follicle attrition

Iatrogenic ovarian injury, particularly following chemotherapy with alkylating agents (e.g., cyclophosphamide, cisplatin) or anthracyclines (e.g., doxorubicin), represents an extreme form of acute oxidative insult. These agents induce a sudden surge of ROS within oocyte and granulosa cell mitochondria, triggering lipid peroxidation, DNA damage and activation of apoptotic or ferroptotic pathways (Trujill et al., 2023). Unlike chronic or progressive disorders, this acute oxidative damage precipitates immediate granulosa death and rapid depletion of primordial and growing follicles. Protective interventions such as NAC, metformin, or HO-1 modulation have shown protective effects in preclinical models by enhancing antioxidant defense (e.g., GPX4, Nrf2, HO-1) and reducing follicular loss (Zhang S. et al., 2023).

**FIGURE 5:**
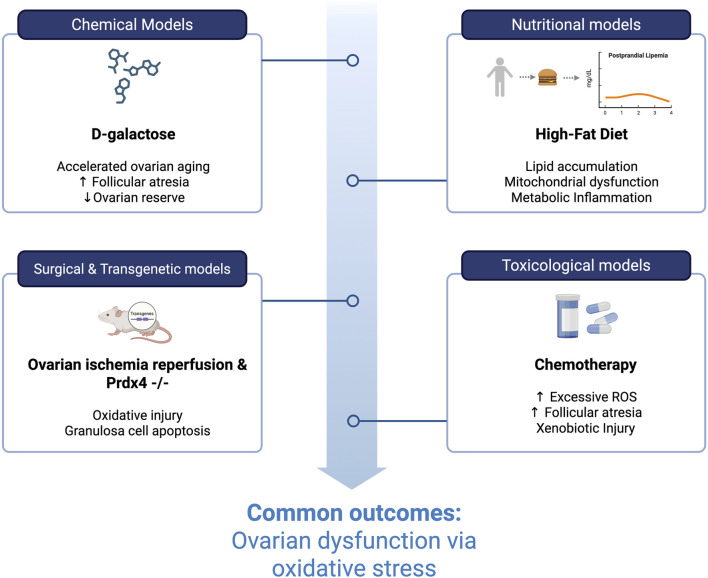
Experimental models converging on oxidative stress-mediated ovarian dysfunction. Distinct chemical, nutritional, surgical/transgenic, and toxicological models induce ovarian damage through diverse upstream mechanisms that ultimately converge on oxidative stress–associated pathways affecting follicular integrity and ovarian function. Created in https://BioRender.com.

Taken together, these disorders share oxidative stress as a common pathogenic denominator, yet differ fundamentally in the timing, intensity, and reversibility of redox perturbations. In PCOS, oxidative stress functions as a chronic, low-grade metabolic disturbance that primarily disrupts follicular maturation and endocrine signaling (Rudnicka et al., 2022). In POI, a rapid and severe oxidative collapse accelerates mitochondrial dysfunction and apoptotic follicular depletion (Shi et al., 2023), leading to a swift decline in ovarian reserve (Houeis et al., 2025). In contrast, iatrogenic ovarian injury is driven by acute and overwhelming oxidative damage, resulting in abrupt granulosa cell loss and follicle attrition (Trujill et al., 2023). These distinctions have direct therapeutic implications, indicating that redox-modulating strategies should be tailored to disease kinetics, with preventive approaches more suitable for chronic conditions such as PCOS (Motta, 2025; Mihanfar et al., 2021), and immediate protective or regenerative interventions required in acute toxic insults (Ruan et al., 2024; Lambertini et al., 2018; Ali et al., 2022). An overview of experimental models converging on oxidative stress–mediated ovarian dysfunction is provided in Figure 5.

## 5 Experimental models of ovarian oxidative stress

Experimental models have been instrumental in elucidating the role of oxidative stress in ovarian dysfunction, enabling the dissection of underlying molecular mechanisms and the assessment of targeted interventions. These models replicate pathological conditions such as POI, polycystic ovary syndrome, and iatrogenic ovarian failure through diverse approaches, including chemical, nutritional, surgical, genetic, and toxicological methods. By inducing oxidative imbalance in controlled settings, researchers have characterized key markers of oxidative damage, mitochondrial dysfunction, and follicular attrition. The following subsections outline the major experimental strategies used to model ovarian oxidative stress, highlighting their relevance in studying disease pathogenesis and testing antioxidant-based therapies (Table 1).

**TABLE 1:** Experimental models of ovarian oxidative stress and their relevance to disease research. The table summarizes commonly used *in vivo* models (chemical, nutritional, surgical, transgenic, and toxicological) highlighting oxidative stress features, ovarian pathologies mimicked, and their utility in studying pathogenesis and antioxidant-based interventions.

Experimental model	Induction method	Observed oxidative effects	Simulated pathology	Animal model	Scientific relevance	References
Chemical (D-galactose-induced aging)	D-galactose administration	↑ ROS, MDA, AGEs, mitochondrial dysfunction, apoptosis, ↑ p16^INK4a	Premature Ovarian Insufficiency (POI)	Rodents (mice/rats)	Mimics ovarian aging; used for testing antioxidants and studying aging-related pathways	Liang et al. (2020), Rostami et al. (2019)
Nutritional (High-Fat Diet, HFD)	High-fat diet feeding	↑ Lipid accumulation, ROS, 8-OHdG, lipid peroxidation, mitochondrial dysfunction, chronic inflammation	Polycystic Ovary Syndrome (PCOS), metabolic impairment	Rodents (mice)	Links metabolism to oxidative ovarian damage; platform for anti-inflammatory and antioxidant therapies	Gonnella et al. (2022), Shen et al. (2023)
Surgical (Ischemia-Reperfusion Injury)	Unilateral/bilateral ovarian ischemia followed by reperfusion	Acute ↑ ROS, localized oxidative damage	Acute ovarian injury	Rodents (rats)	Allows real-time study of acute oxidative injury and regenerative therapy testing (e.g., EVs, stem cells)	Eser et al. (2015), Abdel-Hamid et al. (2023), Güvenç et al. (2024)
Transgenic (Prdx4 knockout)	Genetic deletion of peroxiredoxin-4 (Prdx4^−/−)	↑ Oxidative damage, granulosa cell apoptosis, accelerated ovarian aging under stress (e.g., D-galactose)	Genetically driven oxidative stress	Mice (transgenic)	Reveals role of antioxidant enzymes in ovarian protection and aging	Liang et al. (2020)
Toxicological (Chemotherapy-induced)	Administration of cyclophosphamide, cisplatin, doxorubicin	↑ ROS, mitochondrial dysfunction, follicular apoptosis	Iatrogenic ovarian failure	Rodents (mice/rats)	Models clinical ovarian toxicity; used for testing protective and fertility-preserving agents	Liang et al. (2020), Rostami et al. (2019)
Toxicological (Environmental toxins)	Exposure to polycyclic aromatic hydrocarbons (PAHs) and other xenobiotics	Oxidative damage via environmental toxicants	Environmentally induced ovarian toxicity	Rodents (mice/rats)	Framework for evaluating reproductive risks from environmental exposure and potential protective strategies	Kujjo et al. (2011), Meirow et al. (2001)

### 5.1 Animal models: chemical induction and accelerated aging

Several chemically induced animal models have been employed to investigate ovarian oxidative stress, among which D-galactose-induced ovarian aging in rodents is particularly prominent. D-galactose administration accelerates aging processes by increasing oxidative stress, mitochondrial dysfunction, and apoptosis in ovarian tissue. This model reliably recapitulates human POI, characterized by increased follicular atresia, reduced ovarian reserve, impaired steroidogenesis, and disrupted reproductive hormone levels (Liang et al., 2020; Rostami et al., 2019; Bandyopadhyay et al., 2003).

In rodent studies, D-galactose induces notable oxidative damage markers such as elevated ROS, malondialdehyde (MDA), advanced glycation end products (AGEs), and upregulated senescence-associated proteins including p16^INK4a^ (Liang et al., 2020; Rostami et al., 2019). These findings have provided insight into mechanisms of premature ovarian aging, emphasizing the utility of this model for evaluating antioxidant therapies and molecular pathways involved in ovarian senescence (Liang et al., 2020; Rostami et al., 2019).

### 5.2 Nutritional models of HFD

Nutritional models using a HFD have extensively elucidated links between metabolic dysfunction, lipotoxicity, and ovarian oxidative stress. Rodents fed with HFD develop increased ovarian lipid accumulation, disrupted mitochondrial function, elevated ROS production, and chronic metabolic inflammation, closely mimicking PCOS and metabolic-related reproductive impairment (Gonnella et al., 2022).

HFD-induced oxidative stress models have demonstrated the causal relationship between systemic metabolic derangements and ovarian damage. Mice on HFD exhibit increased markers of oxidative damage (e.g., 8-OHdG, lipid peroxidation), disrupted follicular growth, and diminished fertility outcomes, providing robust platforms to test therapeutic strategies targeting metabolic inflammation and oxidative pathways (Gonnella et al., 2022; Shen et al., 2023).

### 5.3 Surgical and transgenic models

Surgical induction of oxidative stress via unilateral or bilateral ovarian ischemia-reperfusion injury has also been employed. Ischemic models induce rapid oxidative damage by reperfusion-induced ROS, allowing detailed investigations into acute oxidative injury mechanisms, tissue-specific antioxidant responses, and therapeutic efficacy of antioxidants and regenerative interventions such as stem cell-derived EVs (Table 1).

Transgenic mouse models, such as Prdx4^−/−, have offered crucial genetic insights into oxidative stress responses. The loss of peroxiredoxin-4 significantly elevates oxidative damage, granulosa cell apoptosis, and accelerated ovarian aging under stress conditions such as D-galactose administration, establishing genetic links between antioxidant defense impairment and ovarian function (Liang et al., 2020).

### 5.4 Toxicological models: chemotherapy and environmental toxins

Chemotherapy-induced ovarian damage is another key experimental model extensively studied. Chemotherapeutic agents (e.g., cyclophosphamide, cisplatin, doxorubicin) generate excessive ROS, mitochondrial dysfunction, and subsequent follicle apoptosis. These models accurately mimic clinical iatrogenic ovarian failure and facilitate studies into mechanisms and protective interventions (Liang et al., 2020; Rostami et al., 2019).

Additionally, environmental toxin exposure models, including polycyclic aromatic hydrocarbons (PAHs), have been instrumental in understanding xenobiotic-induced oxidative damage and its reproductive consequences. These models highlight the ovotoxic effects of environmental contaminants through oxidative pathways, providing frameworks for evaluating environmental risks and protective strategies (Montano et al., 2025; El-Sikaily et al., 2023).

**FIGURE 6:**
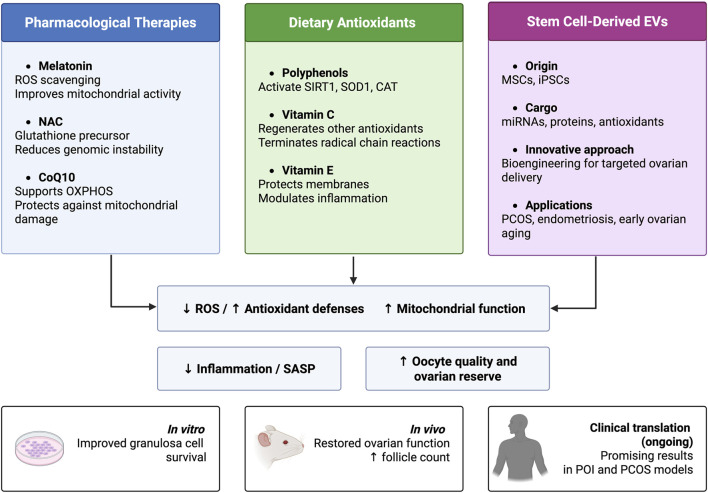
Antioxidant Strategies Against Ovarian Oxidative Stress. The figure illustrates three main antioxidant approaches (pharmacological, dietary, and stem cell-derived EVs) and their shared effects in reducing ROS, enhancing mitochondrial function, and improving ovarian health. Preclinical evidence supports their role in preserving granulosa cell survival, restoring follicular function, and improving outcomes in POI and PCOS models. Created in https://BioRender.com.

A summary of the main animal models used to study ovarian oxidative stress and their key characteristics is presented in Table 1, providing an integrated overview of experimental strategies and their relevance in mimicking human reproductive pathologies. These models are visually categorized by type and mechanism in Figure 6, which highlights their common outcome: ovarian dysfunction driven by oxidative stress.

## 6 Redox-driven regeneration: antioxidant and stem cell-derived strategies to restore ovarian health

Oxidative stress plays a pivotal role in ovarian dysfunction, prompting the development of targeted therapies to restore redox homeostasis and preserve reproductive potential. Antioxidant-based strategies, ranging from pharmacological agents and dietary compounds to emerging regenerative tools such as stem cell-derived EVs, offer promising avenues to mitigate ROS-induced cellular injury and support ovarian function.

### 6.1 Pharmacological antioxidants: mitochondrial rescue and genomic protection

Given the central role of oxidative damage to ovarian aging and dysfunction, antioxidant therapy has emerged as a promising pharmacological strategy tissues (Zhang J. et al., 2019). By detoxifying excess of ROS, such interventions aim to restore redox balance, improve mitochondrial function, and ultimately enhance clinical outcomes. Different antioxidant interventions are currently under investigation, and a wide range of treatment conditions has been described. Some examples are presented below.

Melatonin shows potential in delaying ovarian aging through multiple mechanisms, but available evidence highlights a marked variability in dosages, treatment durations, and clinical outcomes across studies. Preclinical models provide the most robust data: in mice, optimal concentrations ranged from 10^–5^ mol/L, improving oocyte quality and litter size (Zhang L. et al., 2019), to long-term supplementation up to 43 weeks with 100 μg/mL, which increased follicle number, oocyte yield, and fertilization rates (Tamura et al., 2017). Similarly, a 12-month treatment with 10 mg/kg reduced mitochondrial oxidative damage and improved fertility (Song et al., 2016). However, excessive concentrations (1–2 mM) were shown to be toxic, underscoring the importance of dose selection (Adriaens et al., 2006).

Clinical data in humans are more limited and heterogeneous. In perimenopausal women aged 42–49, nightly supplementation with 3 mg melatonin for 6 months significantly reduced LH levels (Bellipanni et al., 2001). In women aged ≥38 years, 2 mg daily for at least 8 weeks was associated with reduced oxidative damage, improved mitochondrial activity, and better IVF outcomes (Tsui et al., 2024). In contrast, a randomized clinical trial in women with diminished ovarian reserve used 3 mg/day only during the ART cycle (approximately 3–5 weeks), reporting higher estradiol levels and improved oocyte and embryo quality without clear effects on pregnancy rates (Jahromi et al., 2017).

Similarly for NAC, available evidence defines clear dose–duration–outcome relationships across models of ovarian aging and ovarian dysfunction. Preclinical evidence indicates that N-acetylcysteine (NAC) improves oocyte quality and delays ovarian aging. In mice, short-term NAC treatment (0.1–1 mM for 2 months) enhanced oocyte and embryo quality, while long-term low-dose administration (0.1 mM for up to 12 months) delayed age-related fertility decline by preserving telomere length and telomerase activity (Liu et al., 2012). Consistently, NAC exerted protective effects in ovarian transplantation models at doses of 150–1,200 mg/kg over 16 days (Amorim et al., 2014). These effects are primarily mediated by reduced oxidative stress, increased telomerase activity, and preservation of follicular development (Wang et al., 2019). Clinically, a meta-analysis of eight randomized trials in women with polycystic ovary syndrome showed that oral NAC (1,200–1800 mg/day for 2–12 months) improved ovulation and pregnancy rates, and an IVF study further reported improved blastocyst quality and reduced gonadotropin requirements in advanced-age women treated with NAC (Li et al., 2022).

Similarly, coenzyme Q10 (CoQ10) has been widely investigated for its role in preserving ovarian function and counteracting age-related reproductive decline. CoQ10, one key component of the mitochondrial electron transport chain, can be found in dietary-derived and synthetic origins. In preclinical models of ovarian failure induced by VCD, cisplatin, or cyclophosphamide, CoQ10 administration at 150 mg/kg/day for 14 days consistently increased follicle numbers and improved oocyte quality, indicating protection of the ovarian reserve (Lee et al., 2021; Özcan et al., 2016; Delkhosh et al., 2019). Clinically, a meta-analysis of 20 trials demonstrated that CoQ10 supplementation significantly increases the number of retrieved oocytes and high-quality embryos, with the most effective regimen being 30 mg/day for 3 months prior to ovarian stimulation, particularly in younger women with diminished ovarian reserve (Shang et al., 2024). In line with these findings, a randomized controlled trial showed that 60 days of CoQ10 pretreatment improved ovarian responsiveness, reduced gonadotropin requirements, and increased fertilization rates (Xu et al., 2018). These effects are mechanistically linked to enhanced mitochondrial function, reduced oxidative stress, and partial rescue of age-related oocyte dysfunction (Ben-Meir et al., 2015; Nie et al., 2023; Brown and McCarthy, 2023).

Despite strong mechanistic support from experimental models, clinical evidence for antioxidant therapies in ovarian aging remains limited and heterogeneous, with most studies relying on small cohorts and surrogate outcomes (Yan et al., 2022; Shang et al., 2024). Variability in experimental models, dosing regimens, and treatment timing limits reproducibility and the definition of safe therapeutic windows. In this context, multiple studies indicate potential risks associated with high-dose or long-term antioxidant supplementation. For instance, Tarín et al. demonstrated that pharmacological doses of vitamins C and E in mice reduced litter frequency, decreased offspring numbers, and increased fetal resorptions (Tarín et al., 2002), while Rutkowski et al. cautioned that excessive intake of synthetic antioxidant vitamins may result in hypervitaminosis and toxicity (Rutkowski and Grzegorczyk, 2012). Consistently, antioxidants have been described as exerting “double-edged effects,” whereby physiological doses may be beneficial, whereas supraphysiological exposure can elicit detrimental cellular responses. Together, these observations raise concerns regarding redox imbalance with prolonged use and underscore the need for adequately powered randomized trials to define optimal dosing strategies and long-term safety profiles (Yang L. et al., 2020; Li X. et al., 2025; Katz-Jaffe et al., 2020).

Key antioxidant compounds and their effects on ovarian function are summarized in the Table 2 below.

**TABLE 2:** Overview of selected antioxidant compounds studied for their effects on ovarian aging. The table summarizes their mechanisms of action, reported benefits, and experimental models used to evaluate their efficacy.

Compound	Mechanism of action	Reported benefits	Experimental models	References
Melatonin	• Potent antioxidant • Upregulates endogenous defenses	• Protects oocytes and granulosa cells • Enhances mitochondrial membrane potential • Improves IVF outcomes in women ≥38 years	Human (IVF patients)	Zhang J. et al. (2019), Tamura et al. (2017), Song et al. (2016), Adriaens et al. (2006), Bellipanni et al. (2001), Tsui et al. (2024), Jahromi et al. (2017)
N-acetyl-L-cysteine (NAC)	• Glutathione precursor • Replenishes thiol reserves	• Reduces oxidative DNA damage • Attenuates telomere attrition	Murine models	Zhang L. et al. (2019), Liu et al. (2012), Amorim et al. (2014), Wang et al. (2019), Li et al. (2022)
Coenzyme Q10 (CoQ10)	• Supports mitochondrial respiratory chain • Reduces lipid/protein peroxidation	• Improves oocyte quality • Enhances ovarian performance in aging models	Murine models	Zhang L. et al. (2019), Lee et al. (2021), Özcan et al. (2016), Delkhosh et al. (2019), Shang et al. (2024), Xu et al. (2018), Ben-Meir et al. (2015), Nie et al. (2023), Brown and McCarthy (2023)

### 6.2 Dietary-derived

Natural dietary compounds play a key role as alternative or complementary agents in antioxidant therapy. Among these, polyphenols are widely studied for their broad biological activities, including the modulation of oxidative stress and inflammation. These effects are mediated through free radical scavenging, the upregulation of antioxidant gene expression, and the enhancement of mitochondrial function. Resveratrol, a well-characterized polyphenol, exerts antioxidant, anti-inflammatory, and anti-aging effects. In the context of ovarian aging, it activates SIRT1-dependent pathways, which are critical for maintaining genomic stability, mitochondrial efficiency, and cellular survival (Pasquariello et al., 2020). Curcumin, another polyphenol, displays similar properties by activating AMPK/mTOR signaling, inhibiting NF-κB, and promoting the expression of endogenous antioxidant enzymes. These mechanisms contribute to its ability to protect ovarian tissue, restore hormone balance, and attenuate oxidative damage in PCOS and POI models (Yang L. et al., 2020; Cheng and He, 2022; Nasiri Bari et al., 2021; Kamal et al., 2021; Yan et al., 2018; Dhiman and Saini, 2025). Quercetin, a flavonoid, has been shown to reduce oxidative stress by upregulating key antioxidant genes such as SOD1, CAT, and GSS, supporting oocyte integrity and ovarian resilience (Zhang J. et al., 2019). Among water-soluble antioxidants, vitamin C (ascorbic acid) functions as a redox cofactor, neutralizing ROS and RNS and regenerating other antioxidants such as vitamin E. Its ability to terminate radical chain reactions makes it an essential component of the intracellular antioxidant defense system (Zhang J. et al., 2019). Vitamin E (α-tocopherol), a lipid-soluble antioxidant, protects cellular membranes from lipid peroxidation by integrating into phospholipid bilayers and interrupting the propagation of lipid radicals. Additionally, it modulates gene expression and immune responses, contributing to oocyte preservation and improved mitochondrial stability in aging ovaries (Agarwal et al., 2008; Zhang J. et al., 2019). Finally, alpha-lipoic acid (ALA), a mitochondrial cofactor and potent antioxidant, contributes to redox homeostasis by regenerating vitamins C and E, modulating NF-κB signaling, and protecting against inflammatory and metabolic damage. Studies have shown that ALA preserves follicular structure and function *in vitro* and may mitigate ovarian dysfunction in reproductive disorders such as PCOS and endometriosis (Di Nicuolo et al., 2021; Ñaupas et al., 2024).

Although dietary-derived antioxidants, particularly polyphenols, demonstrate robust antioxidant and anti-inflammatory effects in preclinical models, their clinical translation is constrained by significant pharmacokinetic limitations. Many polyphenols exhibit low oral bioavailability, rapid metabolism, and limited tissue accumulation, which may substantially reduce their efficacy in human ovarian tissue compared with experimental settings (Gong et al., 2025; Bešlo et al., 2023; Manach et al., 2004). These factors complicate direct extrapolation of preclinical benefits to human reproductive outcomes. Furthermore, clinical studies investigating dietary antioxidants are often heterogeneous in design, dosage, and formulation, with limited follow-up and inconsistent reproductive endpoints (Gong et al., 2025; Irmak et al., 2024). As a result, definitive conclusions regarding their effectiveness in preserving ovarian function or delaying reproductive aging cannot yet be drawn. Large-scale randomized clinical trials are required to define optimal dosing strategies, treatment windows, and long-term safety profiles, particularly in populations without overt reproductive pathology (Gong et al., 2025).

Key dietary-derived antioxidants with demonstrated ovarian protective effects are summarized in Table 3, including their molecular mechanisms, experimental models, and reported outcomes.

**TABLE 3:** Summary of selected dietary antioxidants implicated in the modulation of ovarian aging. The table outlines their mechanisms of action, functional benefits on oocyte and ovarian health, and the experimental models in which they have been evaluated.

Compound	Mechanism of action	Reported benefits	Experimental models	References
Resveratrol	• Activates SIRT1 signaling • Enhances mitochondrial efficiency • Reduces inflammation and oxidative stress	• Improves mitochondrial function • Maintains genomic stability • Protects ovarian tissue	Murine models, *in vitro*	Pasquariello et al. (2020)
Curcumin	• Activates AMPK/mTOR-mediated autophagy • Inhibits NF-κB pathway (via IκBα stabilization, reduced p65 phosphorylation) • Modulates PI3K/AKT/mTOR, PTEN-AKT-FOXO3a, p38 MAPK • Enhances antioxidant enzymes (SOD, CAT, GPx), activates Nrf2/HO-1 and PPAR-γ	• Protects granulosa cells from oxidative apoptosis • Restores ovarian morphology, estrous cycle, and hormonal levels in PCOS models • Reduces ROS/MDA and increases antioxidant defenses (SOD, GSH)	Murine PCOS models (DHEA-induced), oxidative stress (3-NPA), *in vitro* granulosa cells	Yang L. et al. (2020), Cheng and He (2022), Nasiri Bari et al. (2021), Kamal et al. (2021), Yan et al. (2018), Dhiman and Saini (2025)
Lipoic Acid	• Mitochondrial cofactor (PDH, α-KGDH, etc.) • Regenerates GSH, vitamins C and E • Modulates insulin signaling and NF-κB • Inhibits inflammasome and pro-inflammatory cytokines (TNF-α, IL-6, IL-1β) • Upregulates p27^kip1 and suppresses ovarian epithelial tumor cell proliferation	• Prevents follicular degeneration in vitro • Supports follicle development • Antioxidant and anti-inflammatory effects in PCOS, endometriosis, and infertility • Antiproliferative effects on ovarian tumor cells	Ovine ovarian tissue cultures, tumor cell lines	Di Nicuolo et al. (2021), Ñaupas et al. (2024)
Quercetin	• Upregulates antioxidant enzymes (SOD1, CAT, GSS)	• Reduces oxidative damage • Supports ovarian resilience	Murine models	Zhang J. et al. (2019)
Vitamin C (Ascorbic acid)	• Acts as a redox cofactor • Regenerates vitamin E • Terminates ROS chain reactions	• Neutralizes ROS/RNS • Supports antioxidant network	*In vitro*, dietary studies	Zhang J. et al. (2019)
Vitamin E (Alpha-tocopherol)	• Integrates into membranes • Prevents lipid peroxidation • Modulates gene expression and immune response	• Stabilizes mitochondrial function • Preserves oocyte integrity	Murine and human models	Agarwal et al. (2008), Zhang J. et al. (2019)

### 6.3 Stem cell-derived EVs: redox-modulating nanotherapeutics

Stem cell-derived EVs have emerged as functional nanotherapeutics capable of modulating redox balance, inflammation, and tissue repair in the context of ovarian aging (Geng et al., 2023; Zhang et al., 2024). EVs, including exosomes (30–150 nm), microvesicles (100–1,000 nm), and apoptotic bodies (500–5,000 nm), are lipid-based nanocarriers released via distinct biogenetic pathways (Ngo et al., 2025; Fei et al., 2024). Stem cell-derived EVs, particularly those from mesenchymal stem cells (MSCs) and induced pluripotent stem cells (iPSCs), have gained attention for their ability to modulate inflammation, oxidative stress, and tissue repair (Zhou et al., 2023; Firouzabadi et al., 2024). Rather than acting as passive byproducts, EVs serve as functional mediators of intercellular signaling, delivering nucleic acids, proteins, and metabolites to target ovarian cells. Their regenerative and immunomodulatory properties support their potential as nanotherapeutics in the context of ovarian aging.

#### 6.3.1 Bioengineered EVs for targeted ovarian therapy

EVs can be engineered for selective delivery using surface ligands, peptides, or receptor-specific antibodies. Folate receptors, integrins, and hormonal receptors enhance ovarian cell targeting. EVs are used to deliver siRNAs, miRNAs, proteins, or drugs, including dual therapies (e.g., chemo and gene therapy), offering a modular platform for ovarian disease intervention (Alharbi et al., 2024; Zhang and Qin, 2023; Liu et al., 2024).

#### 6.3.2 Therapeutic EVs in ovarian dysfunction: preclinical evidence

##### 6.3.2.1 In vitro studies

Evidence derived from *in vitro* ovarian models provides an important mechanistic framework supporting the feasibility of EV–based strategies for preclinical applications targeting ovarian aging and dysfunction. EVs can be efficiently internalized by granulosa and oocytes (Zhang Y. Y. et al., 2023; Edure et al., 2024). Across representative studies (Zhang Y. Y. et al., 2023; Yang W. et al., 2020; Hung et al., 2017; Gabryś et al., 2022; Yuan et al., 2022; Liu et al., 2023; Lipinska et al., 2025), EVs have been applied using heterogeneous experimental protocols, with doses generally ranging from low µg/mL concentrations (≈3–10 μg/mL) to higher fixed doses around 120–200 μg/mL, and exposure times spanning from short co-culture periods of a few hours (up to ∼14 h) to prolonged treatments covering complete *in vitro* maturation windows according to the specie (approximately 18–38 h). Within this range, beneficial effects have been consistently reported, albeit in a context-dependent manner. More in details, in equine and bovine *in vitro* maturation IVM systems, follicular fluid–derived EVs or small EVs administered at fixed concentrations over defined culture periods improved oocyte nuclear maturation and subsequent embryo developmental competence, particularly under conditions of metabolic stress (Zhang Y. Y. et al., 2023; Yang W. et al., 2020; Hung et al., 2017; Gabryś et al., 2022; Lipinska et al., 2025). In oxidative stress–oriented *in vitro* models, such as primary porcine theca cells, a broader dose–response evaluation revealed that EV treatment (30–120 μg/mL for 24 h) reduced reactive oxygen species accumulation, limited apoptosis, and enhanced steroidogenic activity, underscoring the importance of aligning EV dose and duration with the specific stress paradigm and cellular target (Yuan et al., 2022).

Conversely, murine oocyte co-culture experiments using EVs derived from follicular fluid of older women demonstrated that short-term exposure (≤14 h) to age-associated EV populations can exacerbate oxidative stress, mitochondrial dysfunction, and spindle abnormalities, ultimately impairing oocyte maturation (Liu et al., 2023). Interestingly, Zhang et al. developed an *in vitro* model of aged ovarian follicles to investigate the contribution of extracellular vesicles. They demonstrated that the addition of human umbilical cord–derived MSC-EVs to single follicles isolated from aged mice promoted follicle survival, enhanced granulosa cell proliferation, and improved oocyte quality (Zhang Y. Y. et al., 2023).

Overall, *in vitro* models reveal the strong context dependency of EV-based interventions, showing that EV effects on ovarian cells are highly sensitive to dose, exposure time, EV source, and the applied stress or aging paradigm. These systems are therefore most informative for defining mechanistic boundaries and critical parameters that guide subsequent preclinical testing.

##### 6.3.2.2 In vivo studies

Preclinical *in vivo* studies support a beneficial role of EVs in models of ovarian aging and dysfunction, while simultaneously revealing substantial variability in experimental protocols, dosing strategies, and outcome measures. This heterogeneity is well captured by a recent meta-analysis of 29 studies, which reported significant increases in follicle numbers and ovarian hormone levels following MSC-derived EV administration, but also highlighted a wide range of administered doses (approximately 10–400 µg per dose or 10^6^–10^12^ particles), treatment schedules spanning from single injections to repeated administrations over days or weeks, and variable timing of outcome assessment relative to POI induction (Firouzabadi et al., 2024).

Additional *in vivo* studies further exemplify this diversity. In chemotherapy-induced premature ovarian failure or insufficiency models, EVs derived from human umbilical cord or adipose mesenchymal stem cells have been delivered using different routes, including systemic tail vein injection, intrabursal, or intraovarian administration, employing both single-dose regimens (e.g., 75 μg; (He et al., 2024)) and repeated dosing schedules (e.g., 150 µg weekly for 4 weeks; (Yang et al., 2019)). Despite these differences, convergent functional outcomes have been reported, such as restoration of follicle numbers, normalization of estrous cyclicity and hormone profiles, reduced granulosa cell apoptosis, and improved ovarian architecture, frequently associated with activation of pathways including PI3K/AKT or SMAD signaling (He et al., 2024, Huang et al., 2018, Yang et al., 2019).

Additional variability is observed in ovarian aging models, whether chemotherapy-induced or metabolically driven. In these settings, EVs have been administered either as localized intrabursal injections or systemically at particle-based doses around 1 × 10^8^ particles/mL, with treatment schedules ranging from every 2 days to weekly administrations (Liu et al., 2020) (Li N. et al., 2023). While these interventions consistently report improvements in granulosa cell survival, redox balance, mitochondrial function, and ovarian hormone production, the selected molecular and functional endpoints vary considerably, encompassing follicular activation, angiogenesis, fertility outcomes, and metabolic remodeling (Liu et al., 2020) (Li N. et al., 2023). Moreover, oxidative stress–oriented models using amniotic MSC-derived exosomes further extend this variability, with EVs administered across broad dose ranges and exposure times, yet still demonstrating protective effects on ovarian cells and partial restoration of ovarian function in chemotherapy-induced POI-like ovarian dysfunction (Ding et al., 2020).

*In vivo* preclinical studies demonstrate a convergence of beneficial functional outcomes across ovarian dysfunction models, while also exposing substantial variability in EV sources, dosing regimens, administration routes, and timing. This heterogeneity highlights the need to integrate mechanistic insights from *in vitro* models with optimized *in vivo* protocols to support translational development.

**FIGURE 7:**
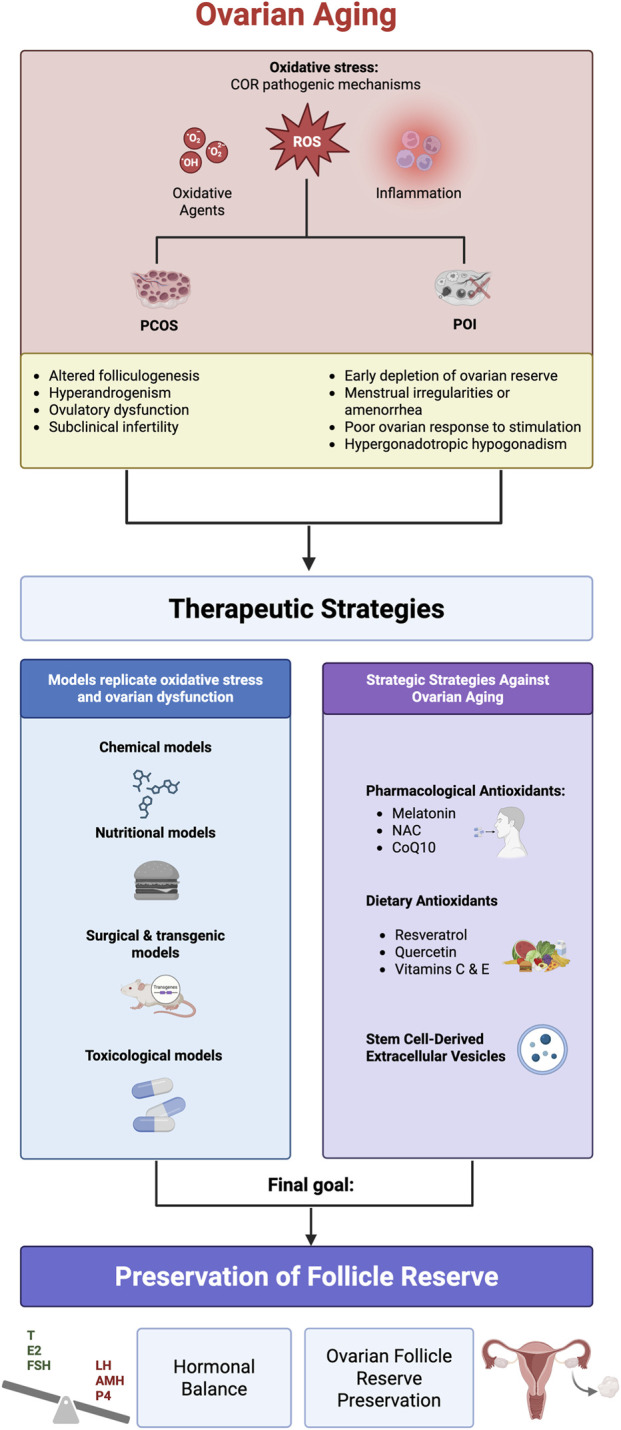
Ovarian Aging and Targeted Therapeutic Strategies for Functional Preservation. Schematic summary of the mechanisms driving ovarian aging (PCOS and POI) and current therapeutic strategies, ranging from experimental models to antioxidant and stem cell-based approaches, aimed at preserving hormonal balance and ovarian function. Created in https://BioRender.com.

To illustrate the growing body of experimental evidence supporting the use of stem cell-derived EVs in ovarian repair, Supplementary Table S1 and Figure 7 provide an overview of preclinical studies demonstrating their efficacy in restoring ovarian function across various disease models.

## 7 Discussion

### 7.1 Limits of preclinical models and translational relevance

While a growing body of preclinical evidence supports the therapeutic potential of redox-targeted antioxidants and stem cell–derived EVs in mitigating oxidative stress and restoring ovarian function, significant translational hurdles remain before these strategies can be applied clinically in women. Most available data derive from rodent models of chemically induced POI, PCOS, high-fat diet exposure, or chemotherapy-related ovarian toxicity. Although these models are instrumental for mechanistic studies, they fail to fully recapitulate the multifactorial and long-term nature of human ovarian aging, which is shaped by decades of environmental exposures, metabolic comorbidities, genetic heterogeneity, and chronic inflamm-aging settings (Wang et al., 2025; Gilmer et al., 2023). Additional limitations include the shorter reproductive lifespan of rodents, the absence of perimenopausal endocrine fluctuations, and species-specific differences in mitochondrial and redox regulation settings (Wang et al., 2025; Gilmer et al., 2023). These factors substantially limit the direct extrapolation of robust preclinical efficacy to human clinical setting as large-scale randomized controlled trials with reproductive endpoints are lacking.

### 7.2 Translational gaps in antioxidant-based interventions

Clinical evidence supporting pharmacological antioxidants such as melatonin, coenzyme Q10, N-acetylcysteine, and dietary polyphenols remains limited and heterogeneous. Existing studies are largely confined to small IVF cohorts or mixed POI/PCOS populations, often characterized by inconsistent dosing regimens, variable bioavailability, short follow-up periods, and the use of surrogate endpoints (e.g., AMH levels) rather than clinically meaningful outcomes such as live birth rates. Consequently, antioxidant interventions have not yet been incorporated into clinical guidelines (Liang J. et al., 2023). Key priorities for translation include the validation of redox and mitochondrial biomarkers linked to reproductive longevity, the design of adequately powered randomized controlled trials with standardized formulations, and the integration of antioxidant strategies with lifestyle or metabolic interventions.

### 7.3 Challenges in standardizing EV isolation, characterization, and validation

For EV-based therapies, major challenges arise from the lack of standardized isolation and characterization protocols. Widely used methods—including differential ultracentrifugation, precipitation-based approaches, and size-exclusion chromatography—yield EV preparations with distinct purity profiles, size distributions, and molecular cargo. Comprehensive characterization of EV identity, including particle size, concentration, surface markers, and cargo composition (miRNAs, proteins, lipids), is not uniformly implemented across studies. Although the MISEV guidelines have improved transparency and rigor in EV reporting, they primarily address minimal reporting standards and do not yet resolve critical issues related to inter- and intra-batch heterogeneity, potency assessment, or clinical-grade validation (MISEV guidelines; (Welsh et al., 2024)).

### 7.4 Regulatory, manufacturing, and GMP-related hurdles

From a regulatory and manufacturing perspective, EV heterogeneity—strongly influenced by cell source, culture conditions, vesiculation stimuli, isolation methods, and storage—represents a central obstacle to reproducibility, comparability, and scalability. Regulatory and GMP-focused analyses emphasize the need for rigorous definition of identity, purity, potency, safety, and stability criteria to generate clinical-grade EV products (Thakur and Rai, 2024). Importantly, GMP-compliant experimental studies demonstrate that the transition from research-grade to clinical-grade EVs requires substantial process modifications, including controlled cell sourcing, closed-system manufacturing, scalable purification strategies, and validated quality controls (Humbert et al., 2025). These changes can profoundly alter EV composition and biological activity, underscoring that manufacturing is not a neutral step but a determinant of therapeutic performance.

### 7.5 Safety considerations and scalability of EV-based therapies

EV-based therapies offer several theoretical safety advantages over whole-cell approaches, including the absence of proliferative capacity, lower immunogenicity, the ability to cross biological barriers, and reduced tumorigenic risk. Consistent with this, systematic reviews report a very low incidence of serious adverse events in EV-based clinical studies to date (Van Delen et al., 2024; Yo et al., 2022; Gowen et al., 2020). Nevertheless, long-term safety data remain limited, particularly with respect to off-target effects, biodistribution, immunomodulation, and potential pro-tumorigenic signaling. In parallel, scalability remains a key bottleneck, as EV production is constrained by low yields and challenges in preserving vesicle integrity during large-scale manufacturing. Emerging strategies—including optimized culture conditions, alternative EV sources, advanced bioreactor systems, and tangential flow filtration—have shown promise, with some approaches achieving up to 100-fold increases in EV yield (Li Z. et al., 2025; Huang et al., 2025; Ng et al., 2022; Debbi et al., 2022). However, these innovations must be integrated with GMP requirements and validated for consistency and functional potency.

### 7.6 Integrative perspective and future outlook

Taken together, both antioxidant-based and EV-based interventions face shared translational limitations rooted in model relevance, standardization, and clinical validation. For antioxidants, inconsistent clinical evidence reflects variability in formulation, dosing, and patient selection. For EVs, the main barriers lie in standardizing isolation, characterization, manufacturing, and regulatory pathways. Bridging these gaps will require the integration of human-relevant models such as ovarian organoids and microphysiological systems, the development of validated biomarkers and potency assays, and the execution of large-scale, well-controlled clinical trials with long-term follow-up. Ultimately, combining redox-modulating strategies with EV-based or nanotherapeutic platforms, guided by patient-specific profiling, may represent a rational path toward extending ovarian healthspan and improving reproductive outcomes.

## 8 Conclusion

Ovarian aging results from cumulative molecular damage that disrupts mitochondrial efficiency and redox balance. Although antioxidant and EVs-based strategies show transformative potential, their clinical realization demands robust human validation, standardized manufacturing, and long-term safety evaluation. Bridging this translational gap through integrative, multidisciplinary research will be essential to develop effective interventions that preserve ovarian function and extend female reproductive health.
