# Supplementary material for: Preventing ovarian aging: from redox-targeted strategies to extracellular vesicle-based therapies
Source: Front Aging. 2026 Feb 24;7:1707614. doi: 10.3389/fragi.2026.1707614 (PMC12971691; doi:10.3389/fragi.2026.1707614)
Supplement: Supplementary file 1 [file Table1.docx]

Supplementary Material

**Supplementary Table 1.** **Preclinical Studies on the Therapeutic Effects of Stem Cell-Derived Extracellular Vesicles in Ovarian Dysfunction Models**. This table summarizes key in vitro and in vivo studies investigating the therapeutic potential of extracellular vesicles (EVs), particularly those derived from mesenchymal stem cells (MSCs), in experimental models of premature ovarian insufficiency (POI), polycystic ovary syndrome (PCOS), and chemotherapy-induced ovarian damage. Details include species, disease model, EV type and source, isolation method, and reported outcomes.

| References | Study type | Species | Model | Type of EV | Source of EVs | Isolation Method | Effect |
| --- | --- | --- | --- | --- | --- | --- | --- |
| (117) | In vivo | Wistar rats | Premature Ovarian Insufficiency (POI) | Exosomes | Amniotic fluid (AF-Exos) | Ultracentrifugation | 1. Increase Follicle Count, decreased atretic follicle count 2. Increased E2 levels 3. Upregulation of cell proliferation proteins (SMAD4 and SMAD6) |
| (111) | In vitro and in vivo | C57/BL6 mice | Chemotherapy-induced premature ovarian failure (POF) | Extracellular vesicles | Human umbilical cord meschenchymal stem cells (HucMSC-EVs) | Ultracentrifugation  Ultrafiltration | 1. Increased proliferation and inhibition of apoptosis of granulosa cells |
| (116) | In vivo | C57B6L/J mice | Premature Ovarian Insufficiency (POI) | Exosomes | Human umbilical cord meschenchymal stem cells (HucMSC-Exos) | Exosome Separation Kit  Differentiation Centrifugation | 1. Decrese in ROS levels 2. Improved ovarian weight and litter size 3. Reduced SIRT4 levels |
| (112) | In vitro and In vitro | ICR mice | Premature Ovarian Insufficiency (POI) | Exosomes | Human adipose mesenchymal stem cells (hADSCs-Exos) | Exosome Separation Kit | 1. Increased hormone levels 2. Increase proliferation rate and inhibition of apoptosis rate 3. Increase in hCG expression 4. Downregulation of SMAD2, SMAD3 and SMAD5 expression |
| (118) | In vitro | Humans | Polycystic ovary syndrome (PCOS) | Exosomes | Adipose mesenchymal stem cells (AMSCs-Exos) | Exosome Separation Kit | 1. Suppression of apoptosis of Cumulus cells in PCOS |
| (110) | In vitro and In vivo | ICR Mice | Premature Ovarian Insufficiency and natural aging model | Exosomes | Human umbilical cord meschenchymal stem cells (HucMSC-Exos) | Differentiation Centrifugation  Ultrafiltration  Ultracentrifugation | 1. Promote follicular activation and development 2. Increased oocyte production and improved oocyte quality 3. Significant decrease of ROS levels in HucMSC-exo treated oocytes and increased mitochondrial membrane potential. |
| (115) | In vivo | C57BL/6 mice | Premature ovarian failure (POF) | Extracellular vesicles | Human umbilical corde Mesenchymal stem cells (HucMSCs-EVs) | Ultracentrifugation | 1. Improved FSH, LH, AMH, E2 and P levels. 2. Increased proliferation of granulosa cells 3. Inhibition of apoptosis of granulosa cells 4. Promotes recovery of granulosa cells through P13K/Akt pathway |
| (113) | In vivo | ICR mice | Premature ovarian insufficiency (POI) | Microvesicles | Human umbilical corde Mesenchymal stem cells (HucMSCs-MVs) | Not detailed. | 1. Increased body weight and number of ovarian follicles 2. Induce ovarian angiogenesis 3. Recovered estrous cycle 4. Upregulation of angiogenic cytokines (VEGF, IGF, angiogenin) |
| (114) | In vivo and in vitro | C57BL/6 mice | Premature ovarian failure (POF) | Small Extracellular vesicles | Embryonic Stem Cells (ESCs-sEVs) | Differential centrifugation  Ultracentrifugation | 1. Recovered serum sex hormones levels 2. Increased number of follicles 3. Decreased number of apoptotic cells 4. Improved proliferation rate of granulosa cells 5. Increased expression of phosphorylated PI3K and AKT |
| (119) | In vivo | C57BL/6 mice | Premature ovarian insufficiency (POI) | Extracellular vesicles | Human umbilical corde mesenchymal stem cells (HucMSCs-EVs) | Exosome Separation Kit  Differential centrifugation | 1. Induced proliferation of Granulosa Cells, increased number of total follicles 2. Increased levels of serum E2 and AMH levels 3. Decreased FSH levels and LH |
